# The Mediating Effect of Coping Style in the Relationship Between Depression and Disordered Eating Among Chinese Female Undergraduates

**DOI:** 10.3389/fpsyg.2019.03011

**Published:** 2020-01-21

**Authors:** Zheng Zheng, Wenyue Han, Yawen Li, Dongyan Wang, Simeng Gu, Fushun Wang

**Affiliations:** ^1^School of Medicine and Holistic Integrative Medicine, Nanjing University of Chinese Medicine, Nanjing, China; ^2^School of Medicine, Jiangsu University, Zhenjiang, China; ^3^Institute of Brain and Psychological Science, Sichuan Normal University, Chengdu, China

**Keywords:** depression, disordered eating, coping style, mediating effect, eating disorder

## Abstract

The aim of the current study was to explore the relationship between depression and disordered eating in female undergraduates and the mediating role of coping style between depression and disordered eating. Self-report questionnaires assessing coping style, disordered eating, and depression were completed in 646 Chinese female undergraduates. The results illustrated that there were obvious differences in disordered eating among the undergraduates with various majors. The disordered eating in female undergraduates majoring in art was more serious than those in other majors. Depression and coping style were effective indicators to predict disordered eating. Moreover, depression could not only directly predict disordered eating, but also predict disordered eating through the mediating effect of coping style. These findings indicate that depression and negative coping style are associated with disordered eating. Coping style could mediate the effect of depression on disordered eating, as these may be an important target for early intervention programs for eating disorder (ED).

## Introduction

Eating disorder (ED) refers to disordered eating caused by the interaction of psychosocial factors and specific cultural factors. According to the classification of DSM-V, ED can be classified into anorexia nervosa (AN), bulimia nervosa (BN), and binge eating disorder (BED) (American Psychiatric Association, 2013). The lifetime prevalence of AN, BN, and BED were 0.9%, 1.5%, and 3.5%, respectively (Hudson et al., 2007). In an eight-year study, 10–13% of young females met the diagnostic criteria for ED in DSM-V (Stice et al., 2013). ED seriously affects patients’ psychosomatic health and has a higher mortality rate as well. 70% of ED patients suffered from comorbidity, such as mood disorders (>40%) and self-harm (>20%), and the risk of suicide was greatly increased (Anna and Linda, 2016). In a meta-analysis study, the weighted mortality rate (i.e., annual mortality per 1000 people) was 5.1% for AN and 1.7% for BN. More importantly, some patients died of suicide (Arcelus et al., 2011). Chinese researchers retrospectively analyzed the clinical data of 51 ED inpatients. The results showed that 33.33% of the patients had attempted suicide (Darong et al., 2002). The early manifestation of ED is disordered eating. Once individuals had disordered eating, such as vomiting after overeating to control their weight and refusing to eat, they often further developed into ED (Ariel et al., 2019). Disordered eating on campus was a common problem (Ward and Hay, 2015). Both college and middle school students were at high risk for disordered eating (Xiaolu and Xiaoming, 2008). From mid-adolescence to mid-adulthood, the proportion of females who used extreme eating behavior to control their weight increased with age (Neumark-Sztainer et al., 2011). In addition to ED, disordered eating is often associated with other problems. For example, sexual risk behavior is closely related to disordered eating. Young females with disordered eating frequently changed their sexual partners and had a higher risk of unprotected sexual behavior (Fergus et al., 2019). Disordered eating was also a potential cause of schizophrenia (Youssef et al., 2018). Moreover, psychopathological symptoms presented a positive correlation with emotional and binge eating (Poínhos et al., 2018).

Eating disorder and disordered eating were associated with emotional state, especially depression (Lazarevich et al., 2016; van Strien et al., 2016). 80% of patients with AN or BN had mood disorders (Godart et al., 2015). Most of the emotional disorders associated with ED are depressive disorder. There might be a two-way relationship between disordered eating and depression (Holm-Denoma et al., 2014). Compared with the patients suffered from simple ED or ED combined with anxiety, ED combined with depression had more complex and severe symptoms (Hughes et al., 2013). Females with self-perceived weight problems had a higher risk of ED among those diagnosed with depression (Küc̨ük et al., 2018). Although the causal relationship between depression and overeating remained to be studied, depression might be the risk, and maintenance factor of overeating (Brechan and Kvalem, 2015).

In addition to depression, there is a significant relationship between disordered eating and coping style. Maladaptive coping strategies were more likely to lead to disordered eating (Megan and Ann, 2019). Avoidant-oriented coping strategies were proved to partially mediate the link between stress and disordered eating (Iolanda et al., 2018). Coping style is also closely related to depression. For example, coping flexibility refers to an individual’s ability to effectively adjust coping strategies according to the situation of stress. Higher coping flexibility was related to the lower depression risk (Kato, 2012). Coping style was related to suicide risk factors (i.e., depression, suicide ideation, and suicide behavior) (Adam et al., 2018). Depressive symptoms increased with the accumulation of stress events and developed corresponding coping strategies (Suzuki et al., 2018). Depressive patients tended to adopt negative coping strategies such as avoidance, especially when they encountered negative evaluation (David et al., 2017). The relationship among depression, coping style and disordered eating has aroused researchers’ great interest. Negative coping strategies were triggered by depression and emotional eating behaviors (Raspopow et al., 2013). Depressive symptoms and avoidance coping strategy to control stress were associated with disordered eating (McGarrity et al., 2019).

In summary, ED is a common kind of psychological disorder that seriously endangers females’ physical and mental health and even lives. Since disordered eating is the early manifestation of ED, if we identify and intervene the individuals who are still in the early stage of disordered eating, we could effectively reduce the incidence of ED. In previous studies, researchers have explored the relationship among depression, coping style and disordered eating, but most of them are the relationship between two variables, while the relationship between the three variables, especially the mediating effect, is still scarce. The relationship among depression, coping style and disordered eating needs to be further explored. In this study, we have two basic hypotheses. Firstly, depression is a vital predictor of disordered eating. Secondly, depression can not only directly predict the severity of disordered eating, but also indirectly affect the disordered eating through the mediating effect of coping style.

## Materials and Methods

### Participants and Procedure

The occurrence of ED shows remarkable gender differences. Since more than 90% of patients with ED are young females, the subjects in this study were female undergraduates from 7 universities in Nanjing, China by cluster sampling. Two classes were randomly selected from each grade of each university. Of 650 eligible students, 646 (99.4%) consented, and took part in the current study, average age 19.4 years (*SD* = 1.1, range = 18–23). 254 (39.3%) participants came from rural area and 392 (60.7%) from urban area. 249 (38.5%) participants majored in science and engineering, 124 (19.2%) in medicine, 74 (11.5%) in art and 199 (30.8%) in liberal arts.

Assessment sessions took place in class during school time, supervised by members of the research team. The survey lasted half an hour and the questionnaires were collected on the spot. The research was approved by the Human Research Ethics Committee of NJUCM. Approval was also granted by each university. Information about the study was provided directly to the participants and informed consent and assent were obtained.

### Measures

The eating disorder inventory (EDI; Garner et al., 1983) measures the severity of disordered eating. Sixty-four items are divided into eight sub-scales, including drive for thinness (DT), body dissatisfaction (BD), bulimia, perfectionism, interpersonal distrust (ID), mature fear (MF), interoceptive awareness (IA), and inefficiency, with varying response options scored 0–5. Higher score indicates more severe disordered eating. We used the Chinese version of EDI revised by Jianping W. The α coefficients of EDI subscales ranged from 0.68 to 0.87, and the cumulative contribution rate of 8 factors was 43.9% (Wei et al., 2005). In the current study, internal consistency α = 0.88.

The Simple coping style questionnaire (SCSQ; Yaning, 1998) contains 20 items assessing coping style which effectively reflect individual’s coping style in the context of Chinese culture. Scores 0 (never) to 3 (always). Items 1–12 belong to positive coping and 13–20 belong to negative coping. If the average difference between positive coping and negative coping is greater than 0, it is positive coping and less than 0 is negative coping. The α coefficient of the full scale was 0.90 and the retest correlation coefficient was 0.89 (Yaning, 1998). In the present study, total questionnaire internal consistency α = 0.76.

The hospital anxiety and depression scale (HADS; Zigmond and Snaith, 1983) examines anxiety and depression symptoms, respectively. The total score of depression or anxiety can be regarded as the severity of symptoms. HADS had good reliability and validity (Olssøn et al., 2005). In the current study, internal consistency α = 0.82.

### Statistical Analysis

All analyses were performed using SPSS22.0. All statistical tests were two-sided and the significance level was set at *p* < 0.05. One-way ANOVA was used to compare differences between groups. Pearson’s correlation was used to examine correlations among disordered eating, depression and coping style. The percentage bootstrap method of deviation correction was used to test the mediating effect.

## Results

### Relationship Between Major and Disordered Eating

The participants were divided into groups according to their majors and the differences of disordered eating were compared. The relationship between major and disordered eating is shown in Table 1. As revealed in the table, there were obvious differences in disordered eating among various majors [*F*_(__3_,_640__)_ = 3.42, *p*<0.05, η^2^ = 0.17]. The disordered eating of female undergraduates majoring in art was more serious than other majors. The results in the table also demonstrated that the DT [*F*_(__3_,_640__)_ = 16.52, *p*<0.001, η^2^ = 0.07], BD [*F*_(__3_,_640__)_ = 5.28, *p* = 0.001, η^2^ = 0.02], and bulimia [*F*_(__3_,_640__)_ = 3.73, *p*<0.05, η^2^ = 0.02] of subjects majoring in art were more remarkable than other majors. See Supplementary Table S1 for the original data of this study.

**TABLE 1:** Comparison of scores of EDI among female college students of different majors.

Major	① (*N* = 249)	② (*N* = 124)	③ (*N* = 74)	④ (*N* = 199)	*F*	*LSD*
EDI	44.81 ± 18.78	42.19 ± 17.94	50.07 ± 23.80	47.66 ± 19.22	3.42^∗^	③>①^∗∗^, ③>②^∗^, ④>②^∗^
DT	4.23 ± 4.10	4.19 ± 4.29	8.13 ± 5.62	5.48 ± 4.47	16.52^∗∗∗^	③>①^∗∗∗^, ③>②^∗∗∗^, ③>④^∗∗∗^ ④>①^∗∗^, ④>②^∗^
BD	11.59 ± 6.19	10.33 ± 5.76	11.82 ± 5.51	13.07 ± 6.51	5.28^∗∗^	③>①^∗^, ③>②^∗∗∗^
Bulimia	2.08 ± 2.65	2.04 ± 2.45	3.31 ± 5.00	2.07 ± 2.63	3.73^∗^	③>①^∗∗^, ③>②^∗∗^, ③>④^∗∗^

*EDI, eating disorder inventory; DT, drive for thinness; BD, body dissatisfaction; ①, medicine; ②, science and engineering; ③, art; ④, liberal arts; ^∗^*p*<0.05 (two-tailed), ^∗∗^*p*<0.01 (two-tailed), and ^∗∗∗^*p*<0.001 (two-tailed).*

### Correlations Among Disordered Eating, Depression, and Coping Style

The means and standard deviation (*SD*) of continuous variables are presented in Table 2. The correlations among disordered eating, depression and coping style are shown in Table 3. As revealed in the table, depression was positively related to disordered eating, whereas coping style was negatively related to disordered eating and depression.

**TABLE 2:** Means and standard deviation (SD) of continuous variables.

Variables	*Mean*	*SD*
EDI (eating disorder inventory)	45.76	19.46
DT (drive for thinness)	5.05	4.60
BD (body dissatisfaction)	11.83	6.19
Bulimia	2.20	2.98
Perfectionism	5.27	3.58
ID (interpersonal distrust)	3.72	3.23
MF (mature fear)	8.26	4.36
IA (interoceptive awareness)	4.72	4.37
Inefficacy	4.70	4.10
Coping style	–0.09	1.35
Depression	5.31	3.07

**TABLE 3:** Pearson’s correlation of various variables.

Variables	1	2	3	4	5	6	7	8	9	10	11
1. EDI	1										
2. DT	0.66^∗∗∗^	1									
3. BD	0.60^∗∗∗^	0.51^∗∗∗^	1								
4. Bulimia	0.57^∗∗∗^	0.41^∗∗∗^	0.17^∗^	1							
5. Perfectionism	0.50^∗∗∗^	0.27^∗∗∗^	0.11^∗∗^	0.31^∗∗∗^	1						
6. ID	0.37^∗∗∗^	−0.08^∗^	−0.01	0.10^∗^	0.09^∗^	1					
7. MF	0.47^∗∗∗^	0.16^∗∗∗^	0.14^∗∗∗^	0.17^∗∗∗^	0.15^∗∗∗^	0.07	1				
8. IA	0.76^∗∗∗^	0.37^∗∗∗^	0.20^∗∗∗^	0.46^∗∗∗^	0.39^∗∗∗^	0.35^∗∗∗^	0.28^∗∗∗^	1			
9. Inefficacy	0.64^∗∗∗^	0.18^∗∗∗^	0.20^∗∗∗^	0.25^∗∗∗^	0.18^∗∗∗^	0.48^∗∗∗^	0.19^∗∗∗^	0.59^∗∗∗^	1		
10. Coping style	−0.28^∗∗∗^	−0.072	−0.03^∗∗∗^	−0.18^∗∗∗^	0.05	−0.31^∗∗∗^	−0.11^∗∗^	−0.30^∗∗∗^	−0.43^∗∗∗^	1	
11. Depression	0.31^∗∗∗^	0.03	0.005	0.15^∗∗∗^	0.08^∗^	0.42^∗∗∗^	0.06	0.35^∗∗∗^	0.48^∗∗∗^	−0.42^∗∗∗^	1

*^∗∗∗^*p* < 0.001 (two-tailed), ^∗∗^*p* < 0.01 (two-tailed), and ^∗^*p* < 0.05 (two-tailed).*

### The Mediating Effect of Coping Style in the Relationship Between Depression and Disordered Eating

The results of correlations analysis showed that the relationship among depression, coping style and disordered eating met the conditions of mediating effect test. The percentage bootstrap method of deviation correction and the method proposed by Hayes (2012) were used to test the mediating effect of coping style on the relationship between depression and disordered eating. The results showed that depression had a significant predictive effect on disordered eating (*β* = 0.3095, *t* = 8.259, *p* < 0.001). The direct predictive effect of depression on disordered eating was still significant when coping style was added as a mediator (*β* = 0.2346, *t* = 5.752, *p* < 0.001). Depression had a significant negative predictive effect on coping style (*β* = −0.4225, *t* = −11.829, *p* < 0.001), and coping style also had a significant negative predictive effect on disordered eating (*β* = −0.1773, *t* = −4.347, *p* < 0.001) (Table 4).

**TABLE 4:** The mediating effect test of coping style.

Regression equation (*N* = 646)		Fit index		Coefficient	
				significance	
		
Result variable	Prediction	*R*^2^	*F*	*β*	*t*
	variable				
Disordered eating	Depression	0.096	68.207^∗∗∗^	0.3095	8.259^∗∗∗^
Coping style	Depression	0.179	139.93^∗∗∗^	–0.4225	–11.829^∗∗∗^
Disordered eating	Coping style	0.122	44.5^∗∗∗^	–0.1773	–4.347^∗∗∗^
	Depression			0.2346	5.752^∗∗∗^

*^∗∗∗^*p* < 0.001 (two-tailed). All variables in the model were brought into regression equation by standardized variables.*

**FIGURE 1:**
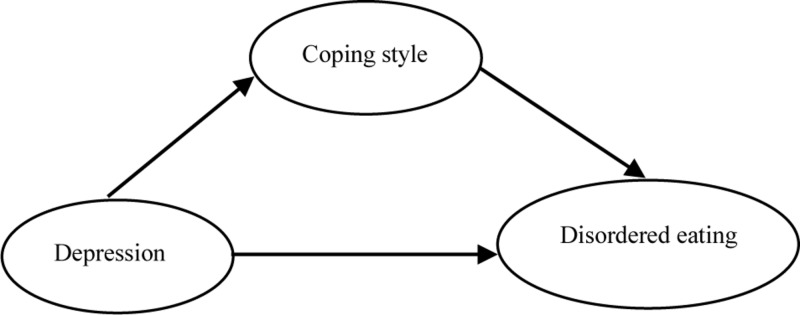
The mediating effect of coping style on depression and disordered eating.

In addition, the upper and lower limits of the 95% confidence interval of bootstrap of the direct effect of depression on disordered eating and the mediating effect of coping style did not contain 0, indicating that depression could not only directly predict disordered eating, but also predict disordered eating through the mediating effect of coping style. The direct effect (0.2346) and intermediate effect (0.0749) accounted for 75.8% and 24.2% of the total effect (0.3095), respectively (Table 5). The mediating effect of coping style on depression and disordered eating is shown in Figure 1.

**TABLE 5:** Analysis of total effect, direct effect, and indirect effect.

	Effect	Boot	Boot CI	Boot CI	Relative
	value	SE	lower	upper	effect value
Total effect	0.309	0.042	0.246	0.391	
Direct effect	0.235	0.041	0.154	0.315	75.8%
Indirect effect	0.075	0.022	0.034	0.118	24.2%

*Boot SE, Boot CI lower and Boot CI upper refer to the standard error of indirect effect estimated by percentile bootstrap method corrected by deviation, the lower limit and upper limit of 95% confidence interval, respectively.*

## Discussion

Our results showed that disordered eating of female undergraduates majoring in art was more severe than other majors, such as medicine, science, and engineering. Previous studies have found that FAT (female athlete triad) was often associated with disordered eating and dancers were more likely to get FAT than runners and figure skaters (Nattiv et al., 2007). Our findings were consistent with previous studies, which showed that disordered eating was closely related to individuals’ occupations.

Eating- and weight-related disturbances were key factors associated with depressive symptoms (Rawana et al., 2016). In this study, we also found a positive correlation between depression and disordered eating. Our study showed that depression could not only directly affect disordered eating, but also indirectly affect disordered eating under the mediating effect of coping style. Coping was a dynamic response to negative life events and subsequent impacts, helping to protect individuals from psychological and physical harms (Annemieke et al., 2019). As early as the end of the 20th century, researchers explored the relationship between stress levels, coping style, and problem-solving ability among bulimic and anorexic individuals. They found that both anorexics and bulimics reported higher levels of stress, lower levels of confidence in their ability to solve problems, a tendency to avoid confronting problems, a reluctance to share personal problems, and feelings of being driven (Soukup et al., 1990). In addition, undergraduates who used maladaptive coping styles showed higher levels of depression and poorer ability to adapt to the environment (Pinkasavage et al., 2015). In this study, we found a consistent result that negative coping style was associated with disordered eating.

This study provides a new perspective to prevent ED by exploring the mediating effect of coping style on depression and disordered eating. Because depression can positively predict disordered eating, when young women have emotional problems, psychological practitioners should pay attention to their eating behavior and evaluate it thoroughly at the same time, so as to identify eating problems early. For individuals with disordered eating, we can focus on alleviating emotional problems to intervene eating problems. Since coping style plays a mediating role between depression and disordered eating, it can effectively alleviate the severity of disordered eating by teaching depressive individuals reasonable coping style and stress management strategies. Focusing on specific positive cognitive coping skills might be an essential way to reduce the frequency of binge eating (Nichole et al., 2012). As females began to adopt adaptive coping strategies, such as seeking support or acceptance from others, they became more confident in using coping strategies and their coping skills were strengthened in consequence (Blevins et al., 2017). In addition, it was useful to prevent and/or reduce disordered eating by teaching students problem-oriented, proactive skills to deal with daily problems related to college life and providing opportunities for repetitive exercises to enhance self-efficacy (Laura et al., 2012).

In the previous hypothesis, we proposed that depression could not only positively predict disordered eating, but also indirectly affect disordered eating through coping style. In this study, the hypothesis was verified. The innovation of this study is to verify the mediating effect of coping style between depression and disordered eating, which provides a theoretical basis for psychological intervention of disordered eating. To sum up, regulating depression and enhancing adaptive coping style can effectively alleviate disordered eating and promote the physical and mental health of female undergraduates, and may further reduce the incidence of ED. The limitation of this study is that we only studied the relationship among depression, coping style and eating behavior, and did not find the causal relationship among them. In addition, through exploratory analysis, we found that coping style plays a mediating role in depression symptoms and disordered eating, but the actual effect of changing coping strategies on preventing ED needs further empirical research.

## Data Availability Statement

The raw data supporting the conclusions of this article will be made available by the authors, without undue reservation, to any qualified researcher. Requests to access the datasets should be directed to ZZ, zzdoctor@126.com.

## Ethics Statement

The studies involving human participants were reviewed and approved by Human Research Ethics Committee of NJUCM. The patients/participants provided their written informed consent to participate in this study.

## Author Contributions

ZZ designed the project and wrote the manuscript. WH performed the questionnaire survey, analyzed the data, and reviewed the literature. YL and DW participated in the revision of the manuscript. SG revised the manuscript. FW revised the manuscript and offered the administrative support.

## Conflict of Interest

The authors declare that the research was conducted in the absence of any commercial or financial relationships that could be construed as a potential conflict of interest.

## Supplementary Material

TABLE S1 Raw data.
